# Evaluation of Multivariable Modeling Methods for Monitoring the Health of Guyed Towers in Overhead Power Lines

**DOI:** 10.3390/s21186144

**Published:** 2021-09-13

**Authors:** Alexandre Schalch Mendes, Janito Vaqueiro Ferreira, Pablo Siqueira Meirelles, Eurípedes Guilherme de Oliveira Nóbrega, Eduardo Rodrigues de Lima, Larissa Medeiros de Almeida

**Affiliations:** 1 Faculty of Mechanical Engineering, Universidade Estadual de Campinas, Campinas 13083-970, Brazil; janito@unicamp.br (J.V.F.); pablo@unicamp.br (P.S.M.); egon@fem.unicamp.br (E.G.d.O.N.); 2 Exploratory Hardware Design Department, Instituto de Pesquisas Eldorado, Campinas 13083-898, Brazil; eduardo.lima@eldorado.org.br; 3 Transmissora Aliança de Energia Elétrica S.A., Rio de Janeiro 20010-010, Brazil; larissa.almeida@taesa.com.br

**Keywords:** IoT, power lines, guyed towers, sensor fusion, Kalman filter

## Abstract

This article proposes a methodology for monitoring the structural stability of each tower of an electric power transmission line through sensor measurements which estimates the different situations that may indicate the need for intervention to prevent the structure collapsing. The extended Kalman filter was adopted to predict the failures, considering sensor fusion techniques such as the displacements of the upper central position of the tower above certain limits. The load of the stay cables is calculated from the natural frequencies, which are determined by the accelerometers connected to the cables. The average value of these forces, which must be higher than a normal limit, were calculated to predict a failure. All guyed towers of a power transmission line thousands of kilometers long will be individually monitored considering the methodology described in this study, which makes this article one of the first relevant research studies in this area. Typically, guyed towers must often be manually inspected to ensure that the stay cables have acceptable pretension to prevent a lack of stability in the transmission line towers.

## 1. Introduction

The collapse of the guyed towers of power transmission lines is often caused by the break or relaxation of the cables due to environmental erosion, especially in old structures. Therefore, the guyed towers of power lines require maintenance due to the time-varying degradation of the strength of the corroded cable during its service life and its corresponding impacts on the reliability of the entire structure. A successful preventive method depends on studying the corrosion characteristics of cables, the fatigue fracture model, as well as the degradation of the cable force under environmental interactions and the load stress as depicted in [1,2].

The major advantage of using guyed towers in power transmission lines is related to the economical savings due to the reduction in structural material, which in some cases can represent more than 20–40% of costs, according to [3]. Their study focused on the research on the structural layout and optimization of high-rise guyed towers. Guyed towers present several advantages regarding costs and weight savings when compared with self-supporting towers and [4,5] studied the importance of the definition of the structure’s initial stress after the assembling process to prevent undesirable behavior from wind influence.

The structural responses of transmission line towers are usually influenced by the action of the wind, foundation degradation, or even due to cable rupture. In [6], a methodology was developed to investigate the dynamic responses caused by cable rupture, which can generate the phenomenon known as a “cascade effect” and in this case, many towers can collapse at the same time, resulting in significant financial losses. The authors also compared the difference between static end dynamic forces in the tower structure.

In the study of [7], an analysis of the tower vibration based on the Internet of Things (IoT) is presented. The authors describe a system which can be divided into the sensing network, data communication and system software. Two towers were considered in their experiment—one guyed tower and a steel tube tower—which were subjected to an impact excitation. There are several types of sensors involved in the study and the measured vibrations were transmitted and analyzed to verify variations that can be caused by the structural degradation of the towers. Thus, it is possible to investigate the health level of the model by comparing the normal values (without abnormalities) with real-time monitored vibrations.

This study proposed a method to prevent the collapse of the guyed towers of a transmission line using modern techniques for monitoring the degradation caused by environmental factors and foundation failures, which are among the less predictable causes of tower collapse. The types of sensors to be considered in this study were analyzed to predict the application of the sensor fusion technique due to the potential of signal condensation from different sensors, which will accurately estimate the position and inclination of the guyed tower.

The originality of the methodology proposed in this article consists of the accuracy in determining the health of each guyed tower from the estimation of the top center position, taking into account the time-variation of the average stay-cable force, which is evaluated based on the measurements of the natural frequencies of the cables. The transfer of the monitored data of all towers to an operation center is based on IoT.

Regarding the results of this study, we can highlight the following advantages:

- The method allows time for the maintenance staff to act;
- The method allows remote measurements;
- The method can accurately monitor and increase system reliability and the quality of service delivery;
- The method reduces preventive maintenance costs.

**Figure 1:**
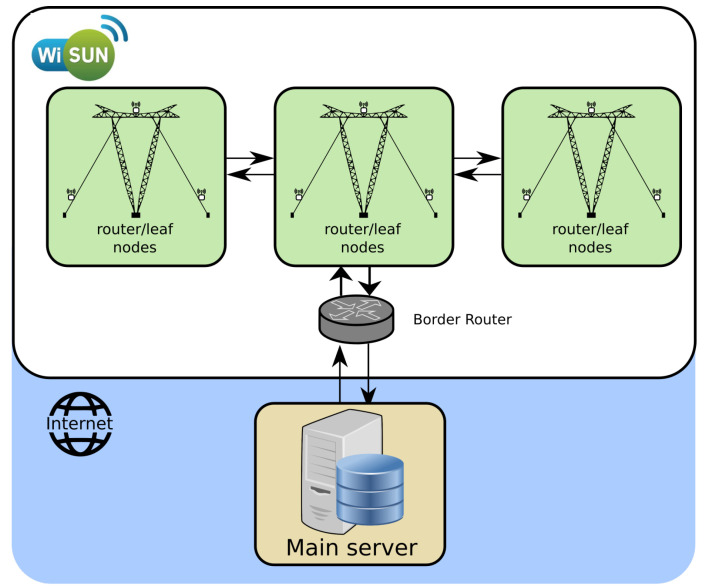
The overall solution framework.

A novel and complete framework was developed for remotely monitoring the structural health of guyed towers. In this way, the framework considers the use of the low-power and lossy networks (LLN). Figure 1 illustrates an implementation of the proposed framework for monitoring the health of power line guyed towers. This consists of sensor watching features at the tower site where the information propagates through an LLN. The data jump from site to site until they reach a data concentrator at the edge of the network.

The overall structural design includes leaf/router nodes and a border node. Router and leaf nodes are restricted to the tower site area and include a set of standard embedded sensors (e.g., accelerometer, gyroscope, thermometer, humidity, and barometer), while specific sensors can be connected on-demand (e.g., load cells, weather station). While the leaf nodes are fixed on the cables, a single router node is positioned at the top of the tower.

Currently, there are many LPWAN technologies, including NB-IoT, LoRa, Sigfox, Wi-SUN, among others. However, in this particular case, the choice for using the Wi-SUN network is due to some reasons:

- Wi-SUN networks are usually meshed networks. This topology allows for multiple redundant connection paths for a network with communicating tower sites, unlike star-based networks;
- Wi-SUN provides high data rates that are consistent throughout the network and presents the lowest latency;
- The current choice uses less power than competitors for listening, thus enabling devices to frequently listen and endure long-term operation;
- Wi-SUN is an open standard, and no company exclusively owns the technology.

Consequently, a more effective corrective action can help the maintenance team improve anchorages as well as increase system reliability and the quality-of-service delivery.

This paper is organized as follows. Section 2 presents the methodology for the dynamic characterization of the stay cables, focusing on the determination of a relation between cable natural frequency and cable forces. Section 3 refers to the definition of the mathematical model for dynamic simulations of the guyed tower. Section 4 shows the displacement estimation of the tower upper center using the extended Kalman filter. Section 5 presents cases of failure simulation executed in the laboratory and the comparison with the expected theoretical results. Finally, Section 6 presents the discussion of the results obtained from a mockup tower at a scale of approximately 1:5.

## 2. Stay Cable Dynamic Characterization

In this section, we will determine the dynamic characteristics of the stay cables used to clamp the prototype tower. For this determination, devices for the dynamic tests were developed in the laboratory of Unicamp—*LabEDin*.

Steidel, as presented in [8], proposed equations for chords and cables which relate different lengths and a natural frequency for a particular cable’s geometry and force. Equation (1) takes into account the flexural stiffness of the cable considering fixed extremities:

(1) $$\omega_{n}=\frac{\pi n}{L}\sqrt{\frac{n^{2}\pi^{2}}{L^{2}}\frac{E\;I}{\mu }+\frac{F}{\mu }}$$

**Figure 2:**
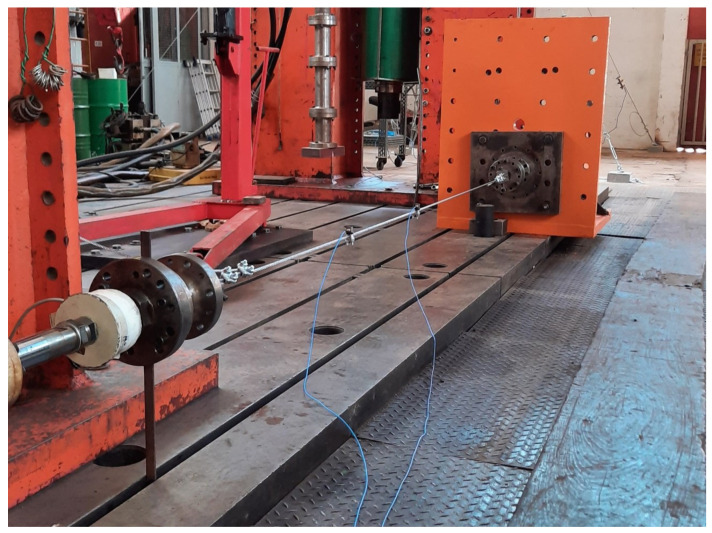
Device used for testing the stay cable of the mockup tower.

**Figure 3:**
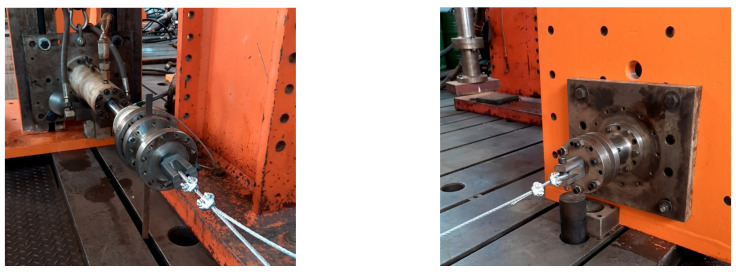
Details of the cable end fixings.

We can observe, that for longer cables, the term that considers the equivalent modulus of elasticity and the polar moment of inertia of the cross section becomes negligible. The cable force, length and linear mass density are the most significant properties. Figure 2 and Figure 3 show the details of the device used for testing the steel cable with a diameter of ϕ1/4”, and Class 6 × 7 with the fiber core is used in the mockup tower. It is possible to observe the positioning of the two accelerometers used to measure the natural frequencies of the cable. They were fixed at exactly the positions of L/2 and L/4, where *L* is the useful cable length. The tests carried out in the cable were important to determine the stiffness, stabilization under loading or unloading conditions and natural frequencies after stabilization phase.

The test consisted of the application of a force through a hydraulic actuator with its subsequent unloading, considering the tensile load sequence in the stay cable with a rupture load limit of 24,500 N. Table 1 presents the applied loads at the mockup tower cable.

Four tests were carried out according to this methodology to obtain the values of the strain and natural frequencies of the cable. The mechanical properties were calculated according to [9]. Table 2 shows the results.

**Figure 4:**
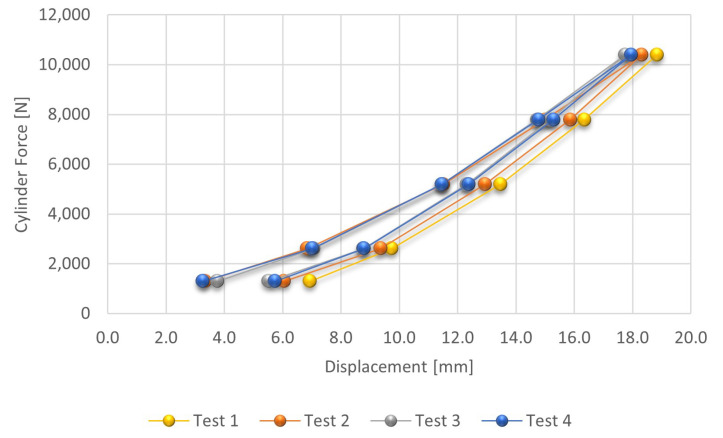
Displacement results as a function of the applied force during the loading and unloading of the mockup tower cable.

The values of the cable loading in Test 1 were not considered in the analysis below due to the occurrence of considerable fluctuations in the values of the measured force. In the sequence, we will present the figures with the test results considering, respectively, the curves for loading, unloading and both curves. Figure 4 shows the variation of the displacements in function of the loading and unloading of the mockup tower cable.

It is possible to observe that there was a small reduction in the displacements from Test 2 to Test 3 and practically no changes from Test 3 to Test 4. This fact indicates that from the third test onwards, the rope stabilized, which shows that despite the ropes being pre-tensioned in the manufacturing process that minimizes this variation, there are still other variations due to the accommodation of the cable fixings.

As well as in the cable loading procedure, during unloading the deformation values stabilize after the third test. Even when taking into account the fact that the elastic limit of the cable may have been exceeded, we can conclude that it presented repeatability for the determination of a relationship between the traction and the natural frequencies of the cable.

**Figure 5:**
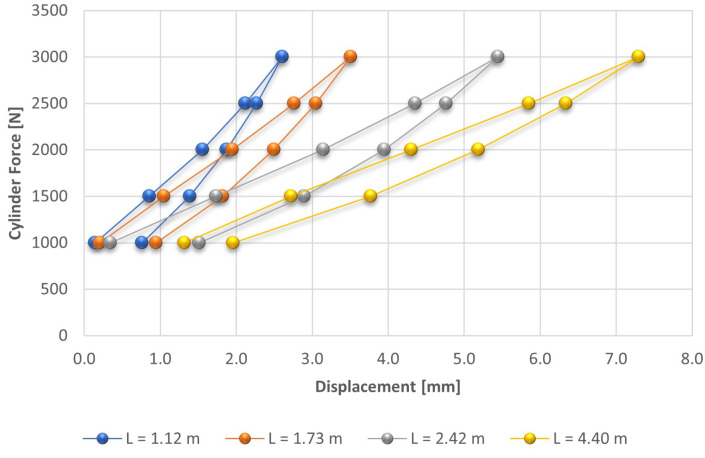
Force at cables with different lengths as a function of the displacement.

Tests were also carried out considering the variation in cable length to determine the relationship between natural frequency as a function of the force applied to the cable. Thus, considering the results from the test with the cable with a length of 2420 mm, three steps of loading and unloading were carried out with cables with lengths of 1120, 1730 and 4400 mm to obtain the cable stabilization. It is important to note that the mockup tower presents stay cables with an effective length close to 4400 mm. The initial length of the cables was determined considering a preload of 600 N. This force was defined in order to eliminate the catenary due to the weight of the cables with sensors. The results are presented in Figure 5.

**Figure 6:**
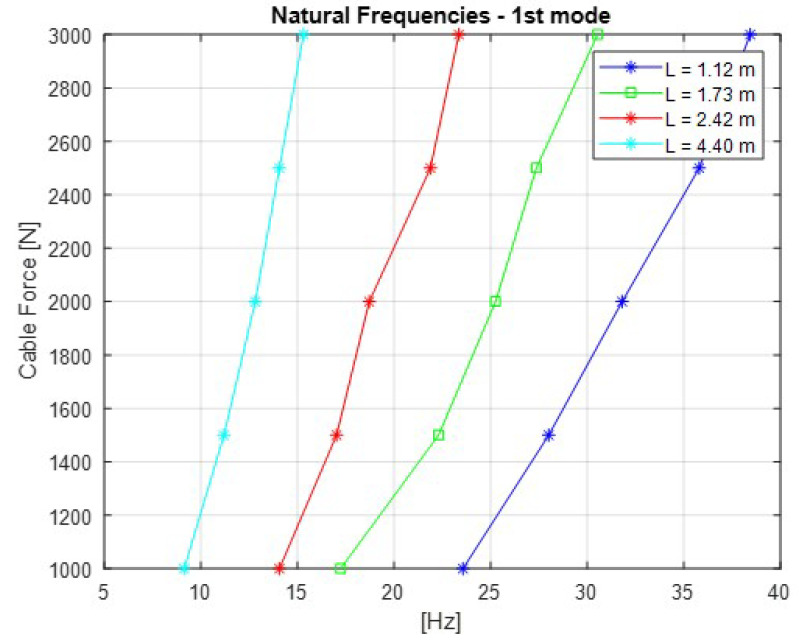
Relationship between the cable force and natural frequencies.

One of the main objectives of these tests was to determine the relationship between the cable traction force and the natural frequencies. Figure 6 shows these results after cable stabilization using an impact hammer. The values are listed in Table 3.

Comparing the measurements of the natural frequencies of the cable during the loading or unloading cycles, it is possible to observe that there is no significant variation in their values.

As expected from the theory, longer cables present lower natural frequencies for the same tractive values. In addition, the frequency variation in the applied load range is smaller for longer cables. Based on the results from the measurements in *LabEDin*, we defined the Equation (2) which calculates the natural frequencies of the stay cable as a function of the applied load. Thus, with the measurements of the accelerometers installed on the cables of the mockup tower, it is possible to determine the load on each cable individually:

(2) $$F_{n}=\frac{0.3027n\sqrt{\frac{F}{\mu }}}{L^{0.718}}$$

where:

$F_{n}$—natural frequency of the cable (Hz);
*n*—mode of vibration (-);
*F*—tensile force of the cable (N);
μ—linear mass density (kg/m^3^);
*L*—equivalent cable length (m).

**Figure 7:**
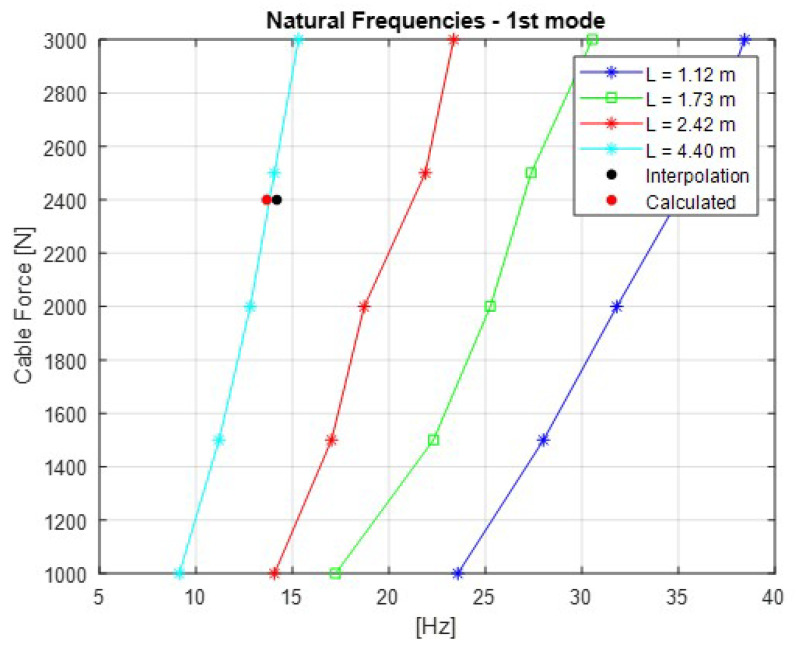
Relationship between the cable force and natural frequencies for the first mode of vibration.

**Figure 8:**
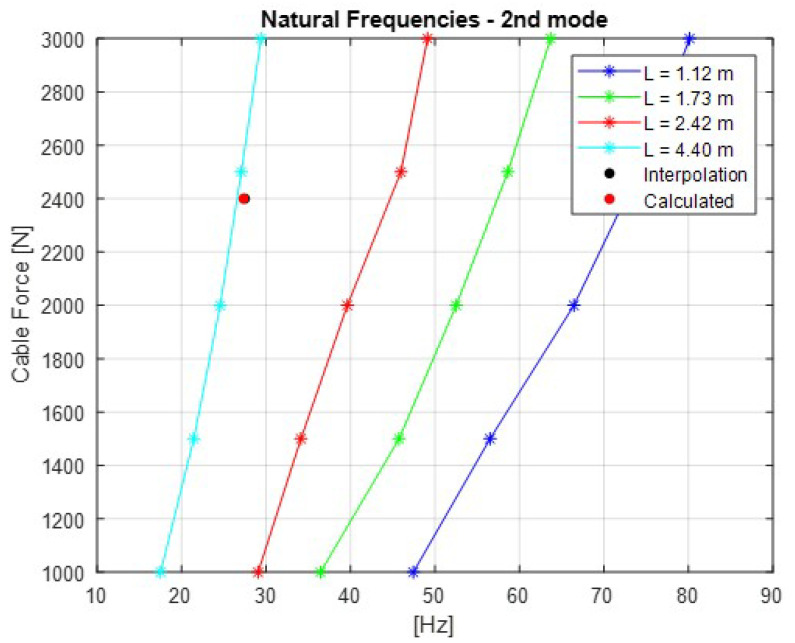
Relationship between the cable force and natural frequencies for the second mode of vibration.

**Figure 9:**
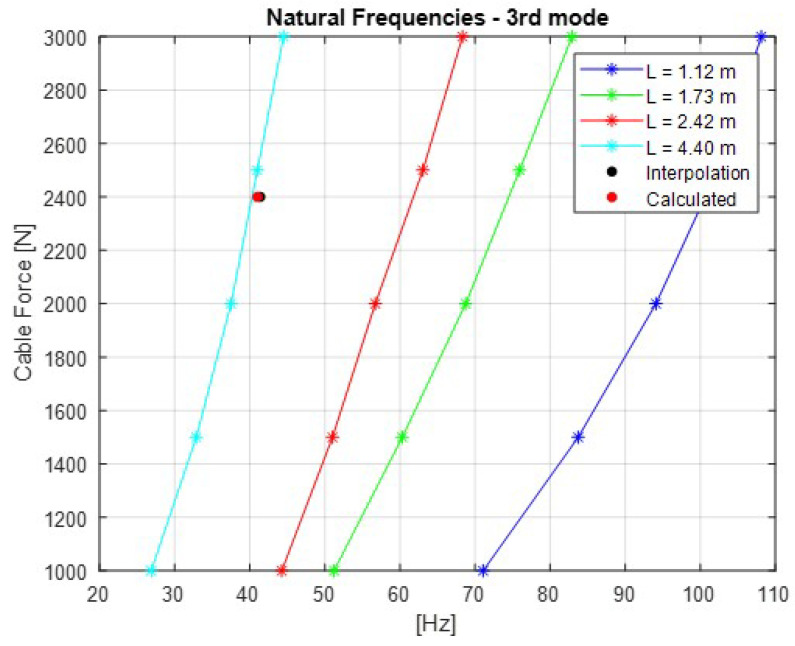
Relationship between the cable force and natural frequencies for the third mode of vibration.

**Figure 10:**
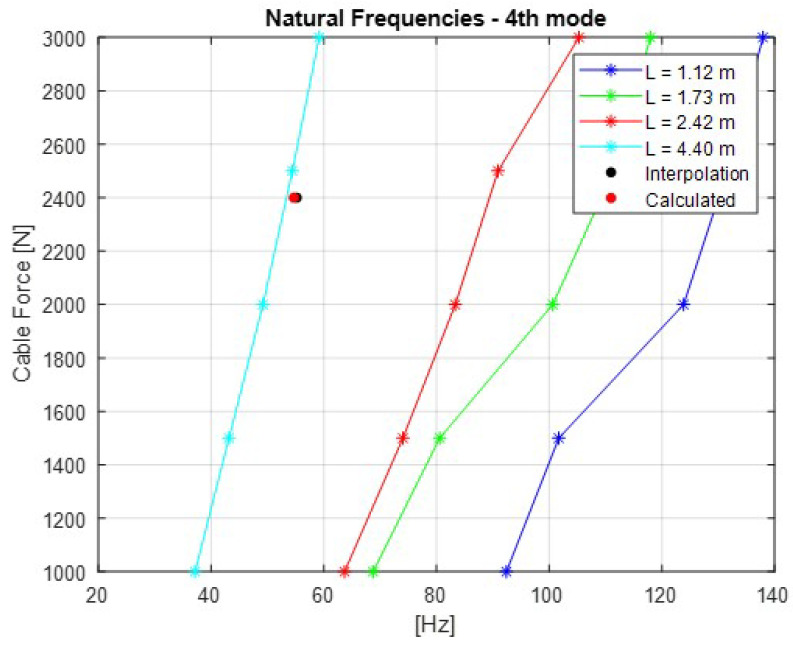
Relationship between the cable force and natural frequencies for the fourth mode of vibration.

**Figure 11:**
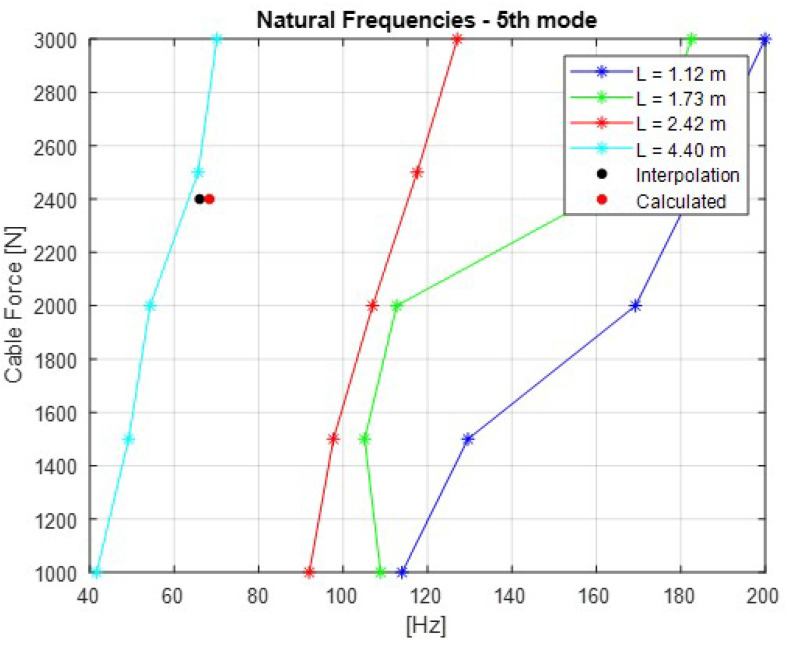
Relationship between the cable force and natural frequencies for the fifth mode of vibration.

The Figure 7, Figure 8, Figure 9, Figure 10 and Figure 11 show the natural frequencies of the four cables tested with different lengths. The 2-D interpolation (black dot) and calculated values with Equation (2) (red dot), which are expected as an example, are also indicated for a cable with a length of 4300 mm and an applied force of 2400 N.

It is possible to conclude that Equation (2) presents good approximation when comparing the 2-D interpolated values with the calculated ones. It is also possible to observe that the calculated natural frequencies are mainly accurate for the lower natural frequencies.

**Figure 12:**
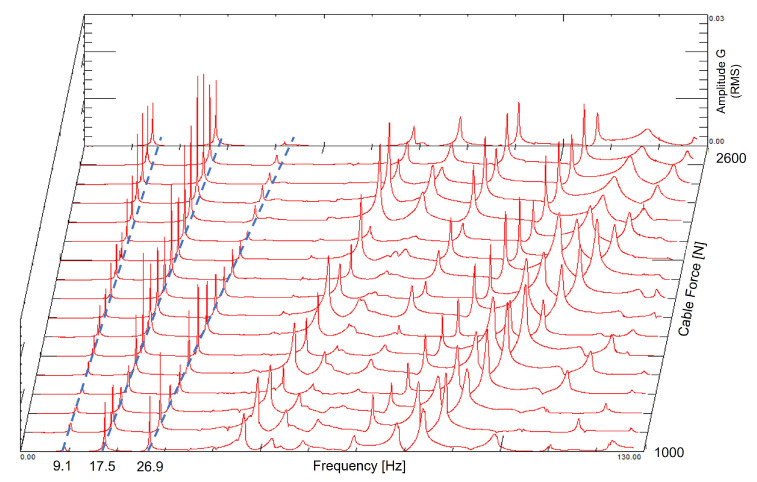
Measured natural frequencies as a function of the cable load (3-D format).

**Figure 13:**
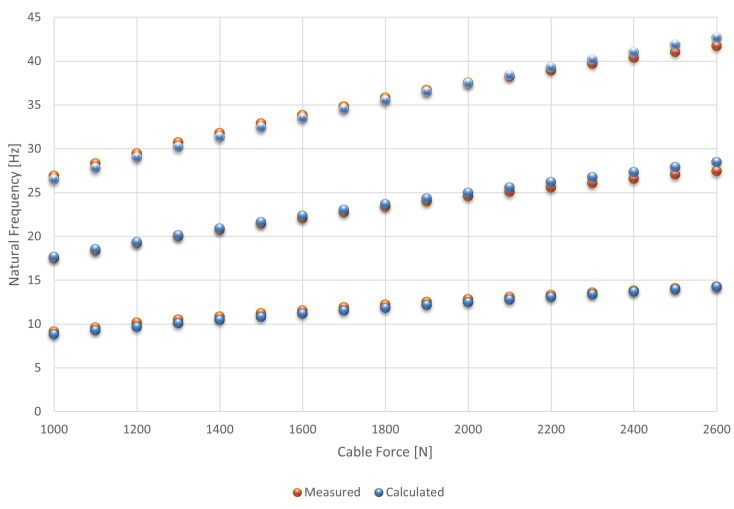
Comparison between the measured and calculated natural frequencies of the first three modes as a function of the cable load (2-D format).

Figure 12 and Figure 13 show the same results in different formats to present the measured natural frequencies. As the accelerometer was positioned close to the node of the fourth mode of vibration, the amplitudes close to 36 Hz are small. By observing Figure 13, it is possible to compare the measured and analytical results of the first three modes of the cable.

Based on the results, we can assume that the use of portable load cells is only important for the initial setup to define the cable natural frequency as a function of the force on each stay. After the assembly, as the natural frequencies are defined, it is possible to remove the portable cells and only proceed with the measured signals from the accelerometers. This proposal leads to a cheaper solution for the predictive analysis of the guyed towers.

The analyses of the accelerations of the cable to define the actuating tractive forces will be used to estimate the displacement of the top center of the tower in the horizontal plane. Thus, it will be possible to identify the existence of displacements in the foundation of the stays that, if not resolved in time through an intervention in the tower, may cause the structure to collapse.

An important conclusion is that since the cable natural frequencies are multiples of a fundamental frequency, it is not complicated to implement an algorithm in the computer code to distinguish the appropriate natural frequencies of the cable, which are not coupled to the structure of the tower. This fact defines with good precision the natural frequency to be used in Equation (2) to calculate the cable force.

## 3. Analytical Model of the Mockup Tower

**Figure 14:**
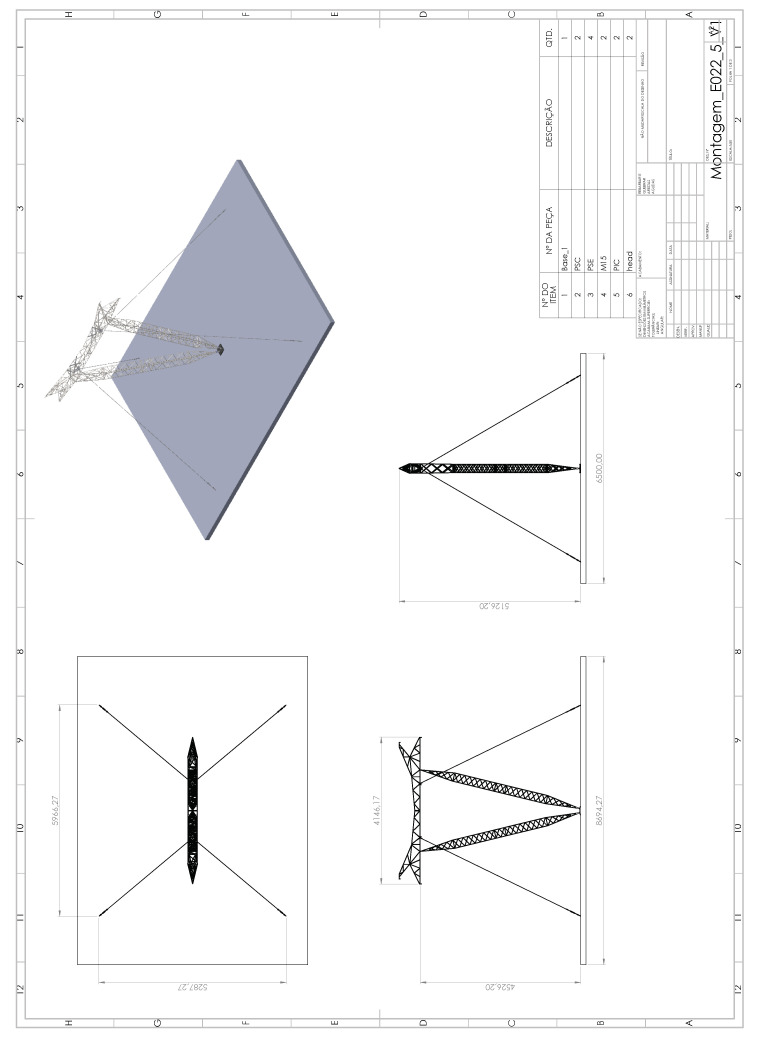
Drawing of the mockup guyed tower.

The transmission line tower structures are presented at different heights that can be selected and installed according to terrain variations. To analyze and reproduce the dynamic responses of a tower, a mockup tower was built with at scale factor of approximately 1:5 in *LabEDin*. Figure 14 shows a drawing of the mockup tower.

A simplified model of the structure of the tower was developed to provide faster evaluations of the dynamic responses from the variations in the force on each cable. This approach renders feasible the application of the Kalman filter for the estimation of tower movements.

**Figure 15:**
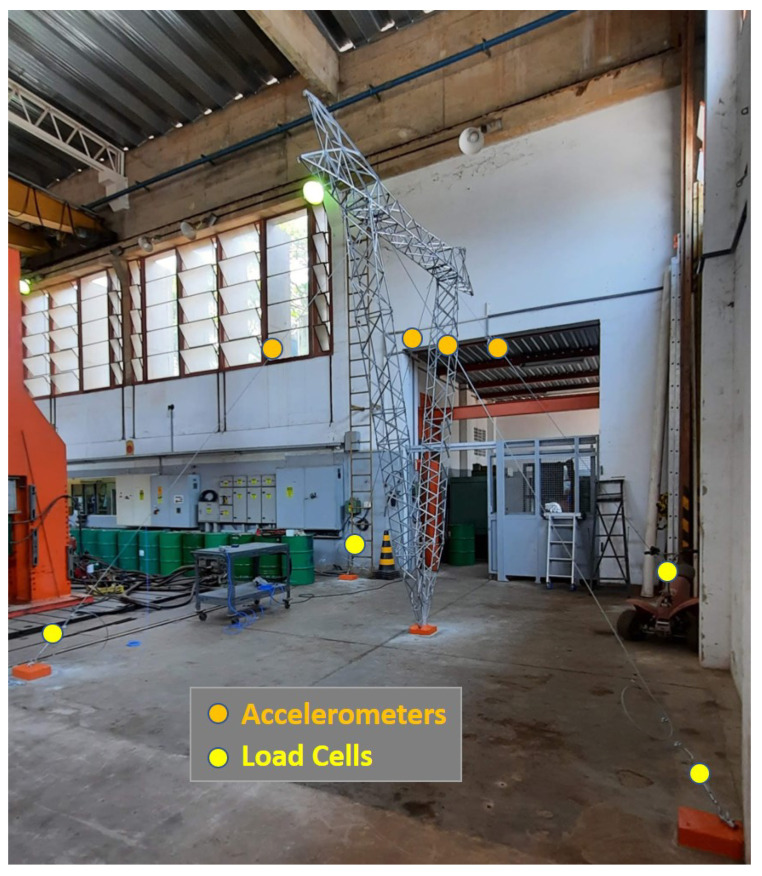
Structure of the stay cable tower assembled in *LabEDin*.

**Figure 16:**
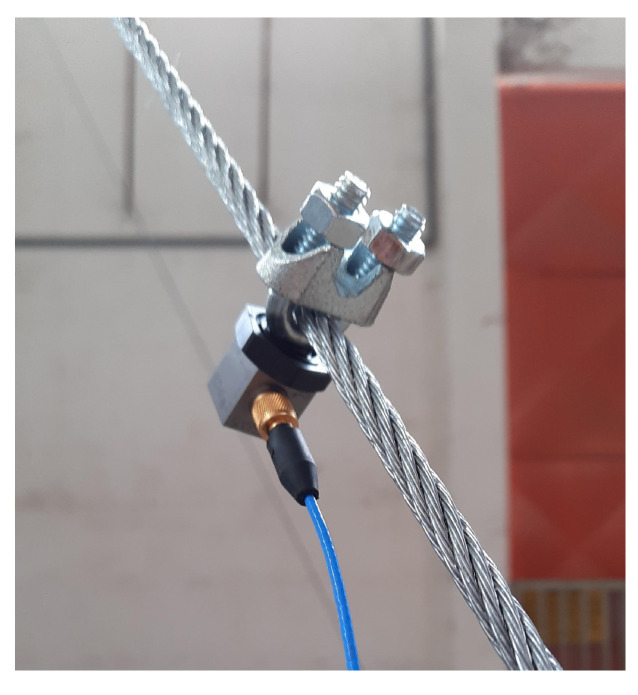
Detail of the fixation of the accelerometers at approximately 65% of the cable length.

**Figure 17:**
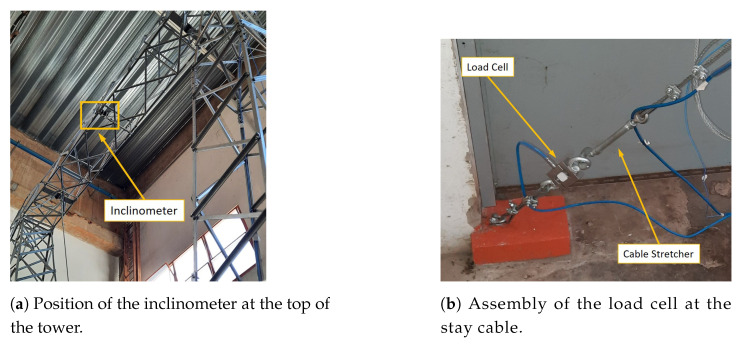
Details of the sensor positioning at the tower in *LabEDin*.

Figure 15 shows the picture of the tower assembled in *LabEDin*, indicating the position of the accelerometers and load cells. Figure 16 and Figure 17 present some details of the devices used in the measurements for monitoring the tower.

**Figure 18:**
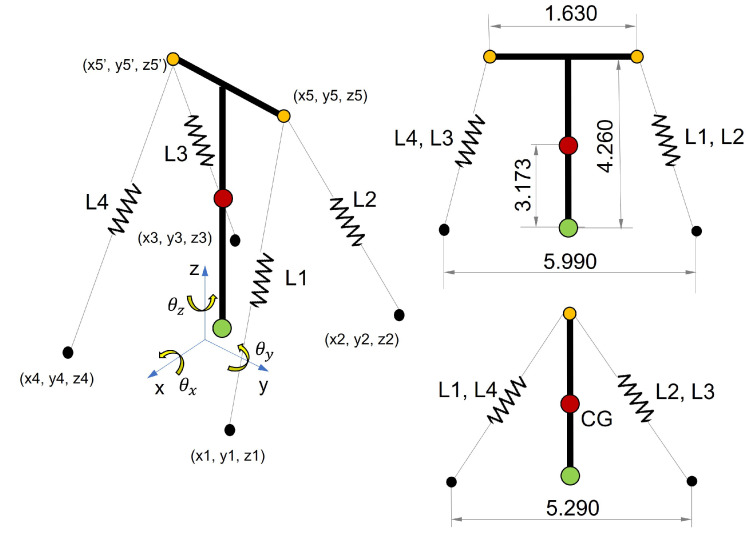
Simplified dynamic model of the tower in *LabEDin*. Dimensions in meters.

Figure 18 illustrates the simplified mathematical model of the mockup tower.

From Classical Mechanics [10], the dynamic equations of the tower model were defined using the energy method, i.e., the Lagrange function, as presented in Equation (3). The angles in the pivot point at the base of the tower are considered generalized coordinates $\theta_{x},\theta_{y},\theta_{z}$, where (i=3) defines the number of degrees of freedom (DOF) of the model:

(3) $$\frac{d}{dt}\left(\frac{\partial L}{\partial {\dot{q}}_{i}}\right)-\frac{\partial L}{\partial q_{i}}=Q_{i}$$

with:

(4) $$L=T-U=T_{v}+T_{\omega }-\left(U_{g}+U_{K}+U_{K_{t}}\right)$$

The kinetic energy is determined as follows:

(5) $$T_{v}+T_{\omega }=\frac{1}{2}m\;v_{cg}^{2}+\frac{1}{2}\left(I_{xx}\;{\dot{\theta }}_{x}^{2}+I_{yy}\;{\dot{\theta }}_{y}^{2}+I_{zz}\;{\dot{\theta }}_{z}^{2}\right)$$

Thus, partial derivations considering small oscillations of the tower are:

(6) $$\frac{d}{dt}\left(\frac{\partial T}{\partial {\dot{\theta }}_{x}}\right)=\left(m\;z_{cg}+I_{xx}\right){\ddot{\theta }}_{x}$$

(7) $$\frac{d}{dt}\left(\frac{\partial T}{\partial {\dot{\theta }}_{y}}\right)=\left(m\;z_{cg}+I_{yy}\right){\ddot{\theta }}_{y}$$

(8) $$\frac{d}{dt}\left(\frac{\partial T}{\partial {\dot{\theta }}_{z}}\right)=I_{zz}{\ddot{\theta }}_{z}$$

Furthermore, with the moments of inertia referring to the center of gravity of the model, the potential energy is defined according to the following equation:

(9) $$\begin{array}{cc} U_{g}+U_{K}+U_{K_{t}}= & m\;g\;z_{cg}\cos\theta_{x}\cos\theta_{y}+\sum_{j=1}^{4}\frac{1}{2}k_{j}{\left(L_{c}-L_{cn}\right)}_{j}^{2}+\cdots \\ & \cdots +\frac{1}{2}k_{t_{x}}\theta_{x}^{2}+\frac{1}{2}k_{t_{y}}\theta_{y}^{2}+\frac{1}{2}k_{t_{z}}\theta_{z}^{2} \end{array}$$

Without the reduction in the accuracy on the results, the partial derivations of the potential energy can be determined according to Equation (10) for small oscillations and disregarding the tower torsional stiffness:

(10) $$\frac{\partial U}{\partial \theta_{x,y,z}}=\frac{\partial U_{K}}{\partial \theta_{x,y,z}}=\sum_{j=1}^{4}k_{j}{\left(L_{c}-L_{cn}\right)}_{j}\;\frac{\partial L_{c_{j}}}{\partial \theta_{x,y,z}}$$

Thus, it is possible to determine the second order differential equations of the motion of the tower according to (11):

(11) $$\left\{\begin{array}{c} \left(m\;z_{cg}+I_{xx}\right){\ddot{\theta }}_{x}-\sum_{j=1}^{4}k_{j}{\left(L_{c}-L_{cn}\right)}_{j}\;\frac{\partial L_{c_{j}}}{\partial \theta_{x}}=Q_{\theta_{x}}\\ \left(m\;z_{cg}+I_{yy}\right){\ddot{\theta }}_{y}-\sum_{j=1}^{4}k_{j}{\left(L_{c}-L_{cn}\right)}_{j}\;\frac{\partial L_{c_{j}}}{\partial \theta_{y}}=Q_{\theta_{y}}\\ I_{zz}{\ddot{\theta }}_{z}-\sum_{j=1}^{4}k_{j}{\left(L_{c}-L_{cn}\right)}_{j}\;\frac{\partial L_{c_{j}}}{\partial \theta_{z}}=Q_{\theta_{z}} \end{array}\right.$$

**Figure 19:**
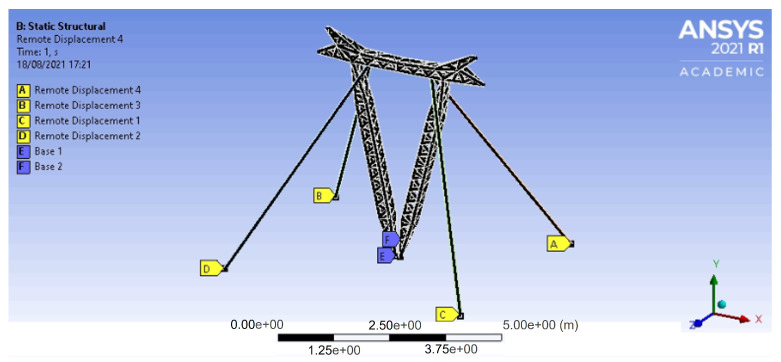
Finite element model of the mockup tower assembled in *LabEDin*.

The required input data of the dynamic characteristics of the tower such as the moments of inertia, mass, position of the center of gravity, etc., was obtained from a finite element model developed to generate simulated data for investigations of the tower behavior. The simplified mathematical model was validated by this FE model, which is presented in Figure 19.

**Figure 20:**
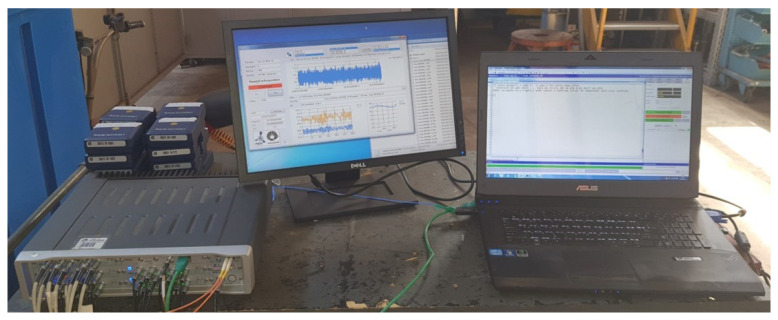
Simens *LMS SCADAS^®^* used for signal acquisition for the mockup tower.

The signal acquisition of each transducer was achieved using *LMS SCADAS Mobile SCR05^®^* (Siemens PLM, Belgium), which has a total of 72 ADC channels. The system was configured with a frequency sampling rate of 256 Hz for a time of 64 seconds, with each sensor generating signals with 16,384 points in time. Figure 20 shows the data acquisition system.

## 4. Introduction to Kalman Filter Concepts

This section will present the main concepts of the Kalman filter algorithm, which were used in the development of the MATLAB^®^ (Mathworks, Natick, MA, USA) code used for subsequent simulations. Additional information can be found in [11,12]. There are several elaborate mathematical concepts that allow the Kalman filter to not only smooth the signals but also estimate the values of different quantities in places of the structure where we do not have access to the instrumentation.

**Figure 21:**
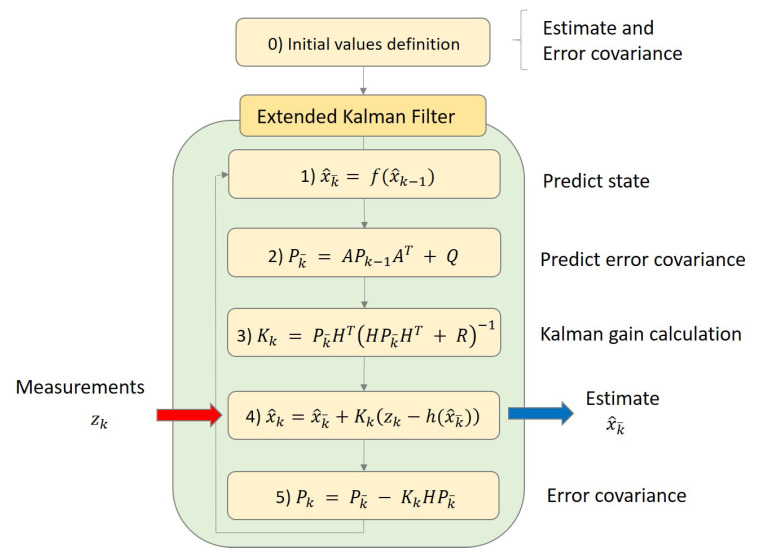
Schematic representation of the extended Kalman filter algorithm.

The application of the conventional Kalman filter for linear systems presents limitations in use, where there are variations in the state transition matrix [A] and/or in the state matrix for the measurement [H], i.e., nonlinear or parameter variant models. Figure 21 illustrates the diagram for the development of the computational code for the extended Kalman filter (EKF) application in the mathematical model.

The state transition matrix varies with rotation angles $\theta_{x},\theta_{y}$ and $\theta_{z}$, which are estimated for each interaction of the algorithm after the calculation of the cable forces from the measured natural frequencies. The variations in the cable force resulted in cable strain variations which were converted into displacements on the X,Y plane at the points where two cables are linked to the tower, i.e., Cables 1 and 2 connected to Point 5 and Cables 3 and 4 connected to Point $5^{\prime }$ (see Figure 18). With the displacements of Points 5 and $5^{\prime }$, it is possible to determine the displacement of the tower’s top center and consequently, the angles in its base position. These three angles correspond to the elements of the state vector, as estimated by the Kalman filter in each interaction.

As mentioned previously, the natural frequencies of the stay cables (in this case, the first mode) were used to calculate the acting force on the stays, which are used to estimate the displacements of the top. Due to the relaxation of the tensioners, there is a decrease in the average force of the stay cables and after the stabilization of the structure, similar forces are observed in all cables.

**Figure 22:**
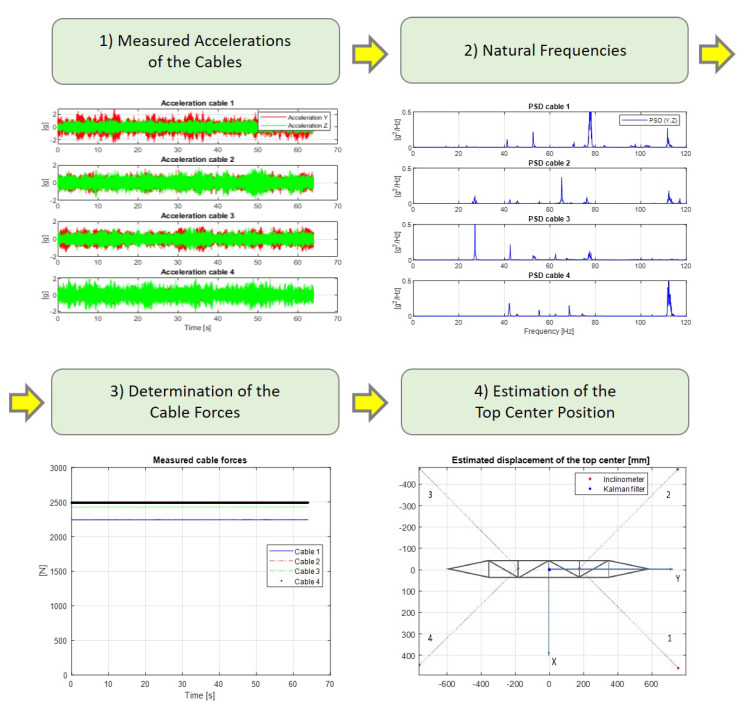
Scheme of the procedure for the estimation of the tower top center position.

The procedure adopted since the acquisition of the acceleration data until the estimation of the position of the tower top center is presented in Figure 22.

To validate and verify the efficiency of the proposed methodology, some experiments were performed in *LabEDin*. A systematic loosening of the cable tensioners was used as a procedure and the position of the tower’s top center was then estimated by the algorithm and compared with the values measured by an inclinometer.

## 5. Failure Simulations in the Mockup Tower

In this section, we will present two cases of failures that can occur in the real towers. The first one considers the relaxation of two stay cables independently, thus the tower inclination increases in function of time. A second case is when two symmetrically opposite stay cables are relaxed. In this situation, the top center of the tower remains at the initial position, but the cable forces decrease which would indicate a possible failure of the structure. In both cases, the sensor fusion technique was used to estimate the position of the top center, considering data from the inclinometer in addition to the cable forces.

**Figure 23:**
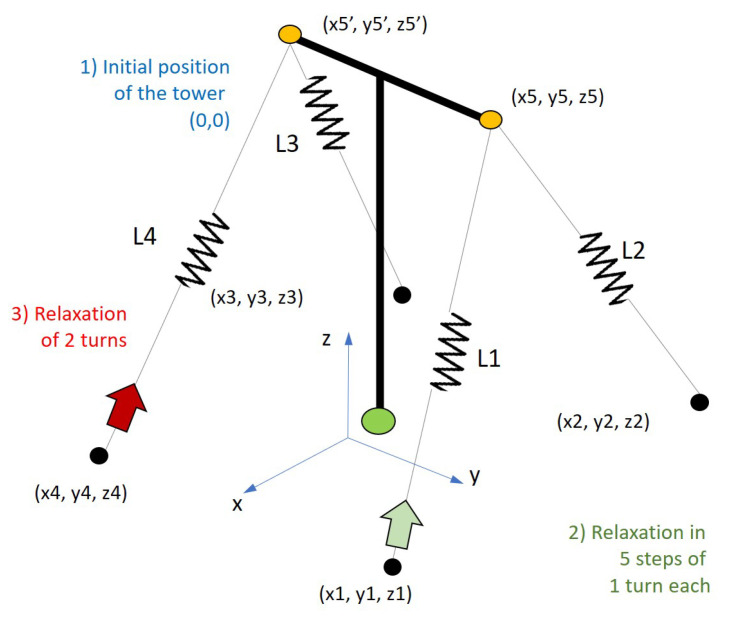
Case 1: Load cases applied to the tower in *LabEDin*, simulating the relaxation of two cables independently.

**Case 1:**Figure 23 shows the intervention sequence applied to the tower’s cable tensioners. Each turn in the tensioner corresponds to a relaxation of the stay cable of 4.20 mm.

**Figure 24:**
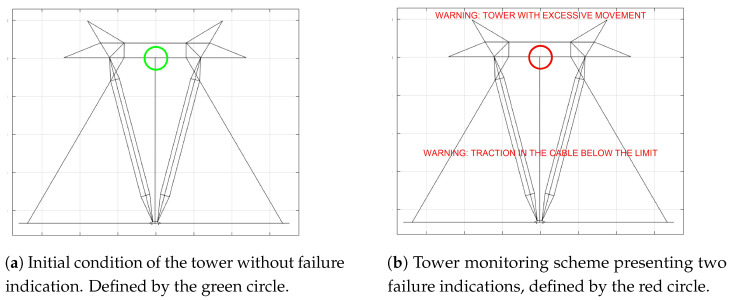
Schematic images of the tower for the status indication.

In Figure 24a, a schematic front view of the tower is shown under the initial conditions, where no fault report is indicated with the cables presenting an average force of 2716 N. The green circle in the middle of the top position indicates that there are no excessive movements of the structure of the tower.

The red circle in Figure 24b indicates that abnormalities occurred in the tower structure and in this case, two failure modes occurred simultaneously, i.e., the average cable force became lower than the defined value (e.g., minimum 1500 N) and the tower top center displacement in the radial direction was higher than the design limit (e.g., maximum 14 mm).

**Figure 25:**
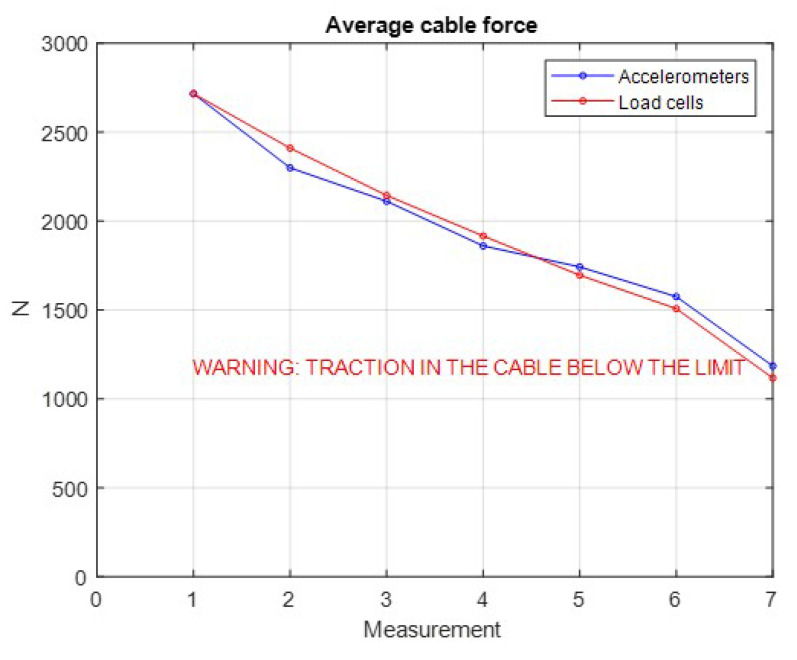
Average cable forces due to manual interventions in the tensioners of Cables 1 and 4.

**Figure 26:**
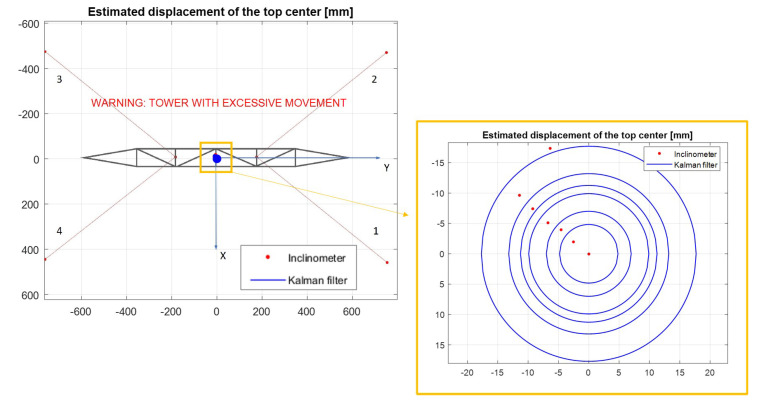
Measured and estimated positions at the top of the tower. Note: tower draft drawing without scale.

Figure 25 shows the variation of the average load of the four stay cables of the mockup tower for each considered load case. In this figure, it is possible to compare the measured values directly from the load cells and the calculated values from the accelerometers. The measured and the estimated values of the displacement of the top due to the relaxation of the tensioners are shown in Figure 26, including a detail of these displacements.

By observing Figure 26, it is possible to see the blue circles which are the estimation of the tower top center position. As mentioned before, the Kalman filter estimates the module of the displacement, however, it is not able to determine its direction. Due to this fact, the actual position of the center can be defined at the circumferences depicted by blue lines, in this case, 7 steps start from position (0,0). The red dots in the picture are the actual position of the top center, measured by the inclinometer.

**Figure 27:**
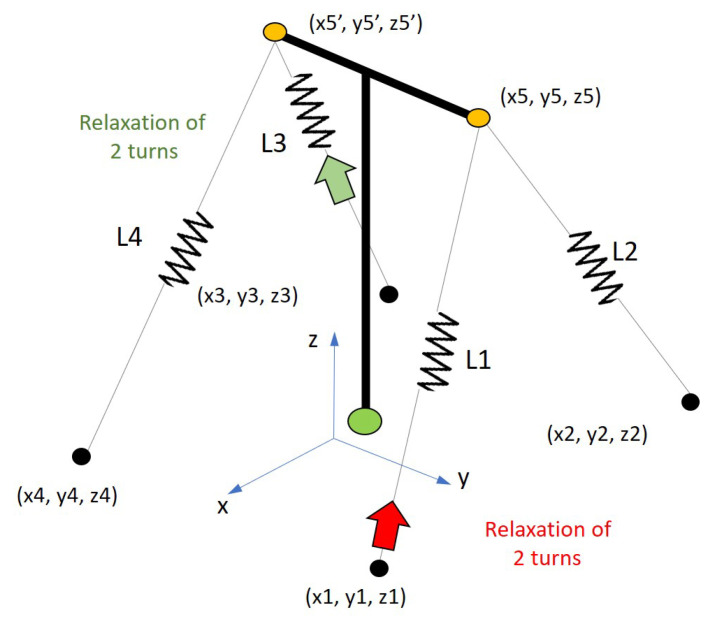
Case 2: load cases applied to the tower in *LabEDin*, simulating the relaxation of two cables simultaneously.

**Case 2:**Figure 27 shows the intervention sequence applied to the tensioners for the second case of failure simulated in the lab.

Here, we assume that two symmetrically opposite cables (L1 and L3) present relaxation at the same time. In reality, this situation is more unlikely to occur compared to the previous case of this article, but it cannot be disregarded.

**Figure 28:**
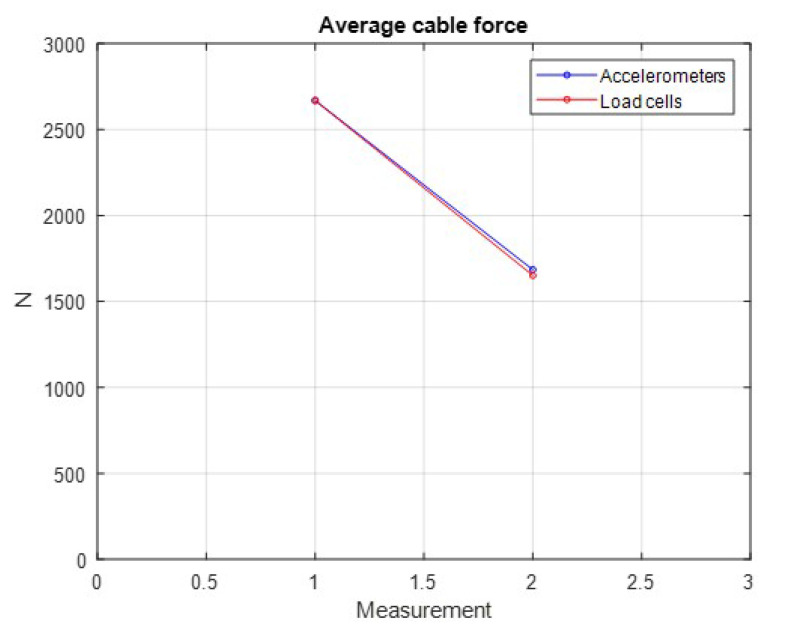
Average cable forces after manual interventions in the tensioners of Cables 1 and 3.

Figure 28 shows the average force at the cables from initial Step 1 to Step 2, where the failure occurred over some time between these two measurements. In this picture, we can also highlight the actual mean forces obtained by the load cells and the calculated values determined by Equation (2).

**Figure 29:**
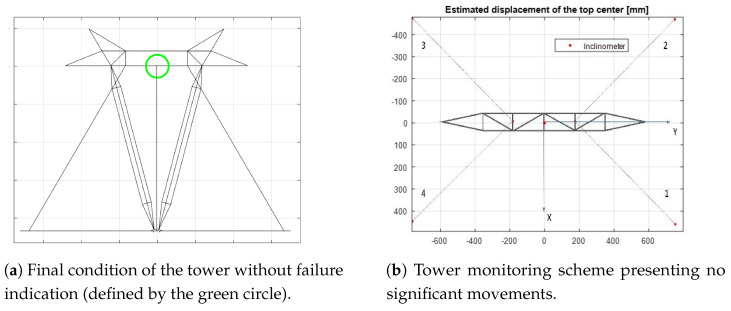
Schematic images of the tower for the status indication at the end of the failure simulation. Note: tower draft drawing without scale.

In this case, no faults were reported, remembering that the minimum average force to indicate a failure was defined as 1500 N and the position of the top center of the tower presents practically no displacements measured by the inclinometer (red dot). Graphically, the indication of the current status of the tower is shown in Figure 29a.

According to the results of this last studied case, we can affirm that it is important to estimate not only the position of the top center, but also the average value of the stay cable forces. Depending on the situation, the tower can be considered stable regarding the estimated position or inclination, but without sufficient cable force, the structure becomes unstable under windy weather conditions. Another assumption in this article is that the tower is designed to withstand severe winds without the occurrence of a large slope of the structure, which returns to the initial condition after the winds end. In addition, electrical insulators promote the decoupling in the vibration transferring from the transmission cables to the tower structure, as can be seen in [13]. Thus, the dynamic analyses of the mockup structure were carried out without considering these components in the laboratory.

It is assumed that there will be no considerable increase in the average force of the cables due to thermal expansion or contraction. Thus, starting from an initial condition with the top center in the position (0,0), all subsequent events that can be considered as failures will always present a decrease in the average cable force. It is important to highlight that whenever there is an intervention in the tower to adjust the forces in the cables and reposition the top center, it is mandatory that the previous measurements are saved or discarded. Thus, the new measurements shall start from this new condition.

## 6. Conclusions

Based on the studies carried out, it was verified that the prototype tower assembled in *LabEDin* presents a stiff and stable structure due to the positioning and fixation of the stay cables. The most significant cause of failure in the tower, which was verified in the lab, is the relaxation of the stay cables. The application of external loads, e.g., the simulation of wind, resulted in small angular amplitude when compared to the displacement of the tower caused by the relaxation of the cable stretchers. In addition, the external loads did not change the average cable force over time and when these loads were removed, the tower returns to its original position. All the effects that were simulated and evaluated in the laboratory are expected to be reproduced in a similar way in real towers of the power transmission line.

Due to the evolution of the methods considered in this study, it was verified that it is not necessary to continuously measure the cable force with portable load cells since it is possible to establish a relationship between the cable natural frequencies and the actuating tractive load. As the determination of the forces will be carried out through the accelerometers fixed in the stay cables, it is possible to disregard the usage of portable cells which reduces the instrumentation costs per each tower and the avoids the use of equipment which can be subject to vandalism.

The inclinometer used in the measurements in *LabEDin* is useful to determine the direction of the tower inclination, since the estimation provided by the Kalman filter only shows the module of the top center displacement. As this estimate was carried out using the average cable force as input, the direction of the displacement is unknown and the fusion of this information with data from the inclinometer allow knowing the exact position of the top center of the tower. However, focusing on the information that reports abnormalities, it is possible to disregard the use of the inclinometer since there is no interest in knowing the direction of the inclination but the absolute value of this inclination. The use of this device can be considered as a redundancy to detect failures due to excessive movement.

A possible divergence that can be found is related to the interference between the resonant frequencies of the transmission lines and the stay cables coupled by the tower structure. This condition must be verified in the actual tower measurements and if this coupling is really observed, the problem can be addressed by specific signal processing techniques. As previously mentioned, based on the conclusions presented in [13], some decoupling of the vibrations from the transmission lines promoted by the electrical insulators is expected.

## Figures and Tables

**Table 1:** Loads applied to the mockup tower cable.

Percentage of the Rupture Load	Load (N)
5.3%	1300
10.6%	2600
21.2%	5200
31.8%	7800
42.4%	10,400
31.8%	7800
21.2%	5200
10.6%	2600
5.3%	1300

**Table 2:** Displacement results as a function of the load applied to the stay cable of the mockup tower (L = 2420 mm).

	Test 1	Test 2	Test 3	Test 4
Load [N]	Displacement	Displacement	Displacement	Displacement
	(mm)	(mm)	(mm)	(mm)
1300	-	3.340	3.760	3.270
2600	-	6.840	7.070	7.020
5200	-	11.520	11.470	11.470
7800	-	15.010	14.740	14.776
10,400	18.840	18.320	17.750	17.960
7800	16.350	15.870	15.210	15.300
5200	13.470	12.940	12.320	12.383
2600	9.740	9.360	8.775	8.790
1300	6.940	6.050	5.520	5.740

**Table 3:** Natural frequencies of the first mode as a function of cable force.

Load [N]	L = 1.12 m	L = 1.73 m	L = 2.42 m	L = 4.40 m
	Nat. Freq. (Hz)	Nat. Freq. (Hz)	Nat. Freq. (Hz)	Nat. Freq. (Hz)
1000	23.56	17.23	14.06	9.13
1500	28.00	22.30	17.02	11.19
2000	31.80	25.25	18.71	12.81
2500	35.79	27.36	21.88	14.06
3000	38.43	30.53	23.35	15.31

## Abbreviations

The following abbreviations are used in this manuscript:

IoT	Internet of Things
LLN	Low-power and lossy networks
LPWAN	Low-power wide-area network
LabEDin	Dynamic Testing Laboratory
DOF	Degrees of freedom
FE	Finite element
ADC	Analog-to-digital converter
EKF	Extended Kalman filter

## Nomenclature

$\omega_{n}$	Natural frequency
*n*	Mode of vibration
*L*	Cable length
*E*	Modulus of elasticity
$I_{p}$	Polar moment of inertia
*F*	Cable tensile force
μ	Linear mass density
$T_{v}$	Translational kinetic energy
$T_{\omega }$	Rotational kinetic energy
$U_{g}$	Gravitational potential energy
$U_{K}$	Linear elastic potential energy
$U_{K_{t}}$	Torsional elastic potential energy
Q	External load
$v_{cg}$	Velocity of the tower’s center of gravity
$z_{cg}$	Height of the tower’s center of gravity
*m*	Mass of the tower structure
*g*	Gravitational acceleration
*I*	Moment of inertia referred to the center of gravity (in x,y,z axes)
θ	Tower angles (in x,y,z axes)
*k*	Stay cable stiffness
$k_{t}$	Tower torsional stiffness at pivot base point
$L_{c}$	Stay cable instantaneous length
$L_{cn}$	Stay cable free length
${\hat{x}}_{k}$	Estimated value at instant *k*
$P_{\bar{k}}$	Error covariance at instant *k*
*A*	State transition matrix
*Q*	Noise covariance matrix of the state transition
$K_{k}$	Kalman gain at instant *k*
*R*	Covariance matrix of the measurement noise
*H*	State matrix for the measurement
$z_{k}$	Measured signal at instant *k*
